# The diurnal pattern of cytokines, chemokines and growth factors in human saliva—a pilot study

**DOI:** 10.3389/fdmed.2024.1420081

**Published:** 2024-11-20

**Authors:** Hajer Jasim, Bijar Ghafouri, Malin Ernberg

**Affiliations:** ^1^ Department of Orofacial Pain and Jaw Function, Public Dental Services, Folktandvården Stockholm, Eastmaninstitutet, Stockholm, Sweden; ^2^ Division of Oral Diagnostics and Rehabilitation, Department of Dental Medicine Karolinska Institutet, Huddinge, Sweden; ^3^ Department of Medical and Health Sciences, Pain and Rehabilitation Centre, Linköping University, Linköping, Sweden

**Keywords:** cytokines, chemokines, diurnal variation, growth factors, periodicity, saliva collection

## Abstract

**Background:**

Understanding of possible periodicity of cytokines, chemokines and growth factors is of great interest and provide valuable information for research into pathophysiological mechanism of inflammatory disease and chronic pain. Significant efforts have been made to identify different analytes in saliva. For precision and accuracy in measurement and interpretation of results, it is crucial to know the source of variability, especially the circadian variation for the analytes.

**Objective:**

The study aimed to analyze circadian variation in 71 inflammatory markers in both unstimulated and stimulated saliva, as well as plasma, from a sample of healthy individuals.

**Methods:**

Ten young adults participated. Unstimulated and stimulated whole saliva were collected at 3-h intervals between between 7:30 am and 7:30 pm. Blood samples were drawn in connection with the first and last saliva collection. All samples were analyzed using the U-PLEX 71-Plex assay.

**Results:**

The analysis showed distinct clustering of the 71 inflammatory mediators between plasma and saliva. Furthermore, differences were also observed between stimulated and unstimulated saliva. The proteins were clustered into three groups that expressed different circadian rhythms. These clusters were stable over time in stimulated saliva but showed significant variability in unstimulated saliva (*P* < 0.05).

**Conclusions:**

These results suggest that time of the day could influence the detection and interpretation of inflammatory markers and collecting saliva samples at consistent times across participants will help control for the natural fluctuations in salivary composition. The results encourage further exploration of salivary diagnostics, particularly in understanding circadian rhythms and localized immune responses.

## Introduction

Saliva contains a variety of molecular and microbial analytes, representing several biological functions that may mirror both oral and systemic health conditions (1–3). With the advancements made in analytical technologies for saliva over the last decades, this body fluid has gained increased attention also for clinical purposes (4). Saliva is presently considered a fluid which can provide information about diseases and not merely as an adjacent to the standard laboratory tests involving for instance blood. There are a growing number of valuable molecules in saliva that have been disclosed, and some of them represent different diseases including cancer (5), autoimmune (6, 7), viral (8–10) and bacterial diseases (11). Saliva collection has also several advantages over blood sampling. It is simple, non-invasive, reduces patient anxiety and discomfort, and facilitates repeated sampling for monitoring. Additionally, saliva collection is safer for health-care providers as it avoids exposure to blood-borne diseases (12), and offers many advantages in terms of storage, shipping and large-scale sampling.

Numerous inflammatory markers have been detected in saliva (13). Cytokines such as interleukin (IL)-1β, IL-6 and IL-4 have been implicated in autoimmune diseases, such as rheumatoid arthritis and diabetes (7). Il-1β appears to be a marker of clinical instability in cardiovascular disorders (14), and IL-10 have been associated with increased risk for myocardial infarction (15). Tumor necrosis factor-alpha (TNF-ά) promotes release of both IL-1β and IL-6 and is linked to chronic disease, including Alzheimer's disease, depression, cancer, chronic pain and multiples sclerosis (13, 16). These and many other substances have been detected in saliva.

Saliva research is limited by variability (13, 17, 18). Important consideration must be given to the effects of saliva collection and its influence on the accuracy of results (19–21). In the last years, remarkable efforts have been devoted on identifying analytes in saliva (19–21). Precision in measurement requires understanding the diurnal pattern (circadian rhythm) of the analytes.

The diurnal pattern refers to the natural, internal processes governed by the circadian clock, which rhythmically coordinates biological activities to ensure they occur at the optimal times, thereby maximizing an individual's fitness (22). It influences the secretion and regulation of various molecules, impacting physiological processes. In particular, the levels of certain hormones (23), cytokines, chemokines, and growth factors vary with the circadian rhythm (24), emphasizing the importance of time in sample collection. The advancement and success of salivary research is attributed to reducing the variability.

The aim of the current study was to analyze the diurnal periodicity for a panel of 71 cytokines, chemokines, and growth factors in whole unstimulated and stimulated saliva in a sample of well-defined healthy individuals. A second aim was to observe the association in these analytes between saliva and blood.

## Materials and methods

### Participants

Healthy participants of both sexes aged 22–32 years were recruited to the study by advertisement. To ensure eligibility, potential study participants underwent a two-stage screening process. Initially, they were screened via telephone, followed by an in-person screening during their first visit. The exclusion criteria included any reported diseases or ongoing pain problem, mental health conditions, regular use of any medication, and oral complaints such as oral dryness or mucosal lesions. Females who were pregnant or actively attempting to conceive were also excluded from the study. Participants reporting pathological levels of psychological distress according to validated questionnaires, were likewise excluded. If oral examination indicated less than 22 teeth, extensive prosthodontics, current orthodontics or endodontic treatment, poor oral hygiene (plaque index > 30% and/or periodontal disease (25), hyposalivation (26), oral diseases, mucosal lesions or oral inflammation they were excluded from further involvement in the study.

All participants underwent a comprehensive clinical examination and completed validated written questionnaires approximately one week before the trial, as detailed below. Participants were requested not to eat, drink or brush their teeth prior to the first collection (fasting), and not consume alcoholic beverages 24 h prior to collection. They were also instructed to keep a detailed food log 24 h prior to and during the day of collection. A brief oral interview was carried out by the examiner at the time of collection to ensure that they had followed the instructions, which all had. Saliva and plasma samples were then collected. During the day of collection, participants were asked not to eat, drink or brush their teeth 1 h prior each collection time-point.

The study was conducted at the Department of Dental Medicine at Karolinska Institutet between December 2014 and September 2015. All participants received information regarding the objectives and procedures of the study and gave their informed written consent before enrolment. The study was approved by the Regional Ethical Review Board in Stockholm, Sweden (2014/17-31/3) and followed the guidelines according to the Declaration of Helsinki.

### Clinical examination and questionnaires

All participants underwent a profound general dental and oral examination. During the clinical examination participants were checked for decayed teeth, dental and oral inflammation/infections, mucosal lesions and oral hygiene (measured as plaque index, pocket probing length). Occlusion, previous dental treatments, erosions, and attrition damage on teeth were also recorded. Participants were also evaluated using the Swedish version of the Diagnostic Criteria for Temporomandibular Disorders (DC/TMD) Axis I and II. This evidence-based screening protocol was employed to identify TMD signs that might not be evident during the interview (27).

The following brief screening instruments included in the DC/TMD axis II (27) were used to evaluate symptoms of depression, somatization, anxiety, stress, jaw function and oral health: the Patient Health Questionnaire (PHQ-9 and PHQ-15), the Generalized Anxiety Disorder scale (GAD-7), the Perceived Stress Scale-10 (PSS-10) and Jaw Functional Limitation Scale (JFLS).

### Saliva collection

Unstimulated and stimulated whole saliva were collected from all participants. Prior to each saliva collection session participants were instructed to rinse their mouth with 10 ml of distilled deionized water for 30 s to remove debris and moisturise the mucosa and thereafter rest for 5 min.

Partly unstimulated whole saliva was first collected as described earlier by the authors (19, 20). Participants were instructed to sit upright with their head slightly titled forward and a polypropylene tube was used to collect saliva during passive drooling. Five minutes afterwards stimulated whole saliva was collected using paraffin gum (Orion Diagnostica, Finland). For pre stimulation, the participants were instructed to chew the gum until it was smooth and flexible. After 60 s of pre stimulation, the participants were asked to swallow the saliva present in the mouth. Subsequently, whole saliva, stimulated by the same piece of paraffin, was collected for 3 min, and salivary flow rate was measured.

Saliva samples were collected during five times during the same day at the exact same circumstances. Samples were collected at 7.30 am; 10.30 am; 1.30 pm, 4.30 pm and 7.30 pm. All participants were asked to come fasting to the first sample. Between each session participants were asked to not eat/drink at least 1 h prior to sample collection.

**Figure 1:**
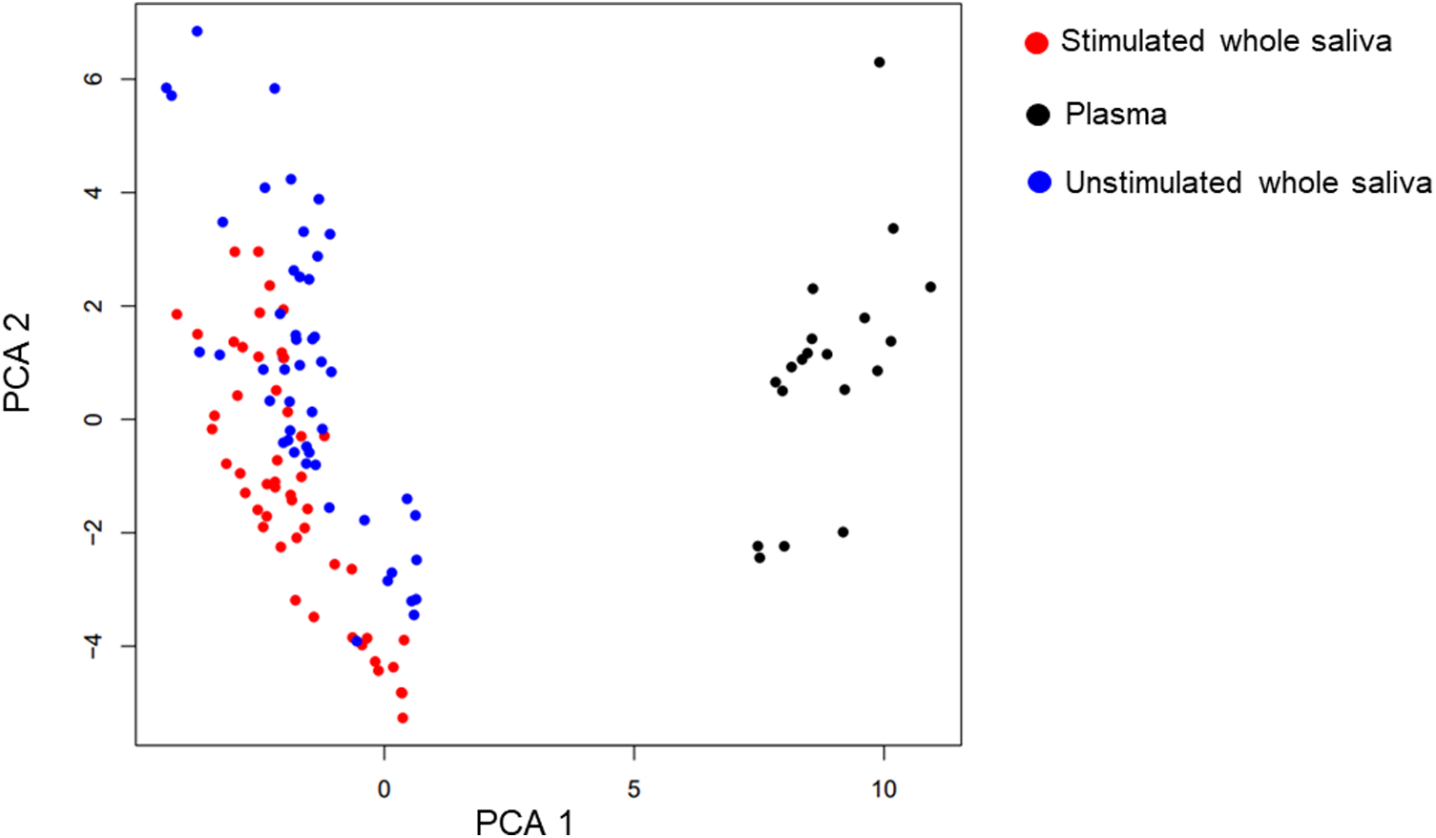
UMAP and PCA. Uniform Manifold Approximation and Projection (UMAP) is used to graphically cluster the proteins irrespective of timepoint. Neither the UMAP graph nor the Principal component analysis (PCA) take into consideration the temporal aspect of the measurements. The analysis is indicative of the plasma samples (black) clustering very differently than the two saliva samples (blue and red). However, even the two saliva sample types cluster clearly with both the methods. The graphs are coloured both showing the sample type and the timepoint. The time of sampling does not seem to be as important to the clustering as the sample type.

To prevent degradation of sensitive proteins all samples were collected on ice in precooled polypropylene tubes. Immediately after collection a Protease Inhibitor Cocktail (Sigma Aldrich v/v 1:500) was added. All samples where then centrifuged at 1500xg for 15 min at 4°C to remove debris. The supernatant (upper 2/3) of each sample was fractionated into 100 µl aliquots and frozen at −86°C until analyses.

### Plasma collection

In connection with the first and the last saliva sample, venous blood samples were collected in 8.5 ml EDTA PPT tubes from all subjects. The sample was mixed gently for 1 min and then immediately placed on ice for 30 min. The samples were then centrifuged at 1000 × g for 15 min at 4°C, and the upper 2/3 of the plasma was stored as aliquots at −70°C until analysis.

### Multiplex immunoassay

A commercially available panel of 71 pro- and anti-inflammatory proteins (including cytokines, chemokines, and growth factors) (U-PLEX, Meso Scale Discovery, Maryland, USA) was used (see Supplementary Table 1). After thawing, the samples were centrifuged briefly and analyzed according to the manufacturer protocol. Briefly, the antibody-samples were mixed with corresponding linkers and 50 µl were pipetted to the 96-well (10 spots) plates, which the antibodies bound to and the plate was coated with the antibodies on gentle shaking at 4°C overnight. Then the unbound antibodies were washed out using 200 µl washing buffer (PBS, 0.05% Tween-20) 3 times. Plasma or saliva samples were diluted 1:2 for almost all substances except for Chitinase-3-like protein 1 (YKL40), Macrophage Inflammatory Protein 5 (MIP-5), and Macrophage Migration Inhibitory Factor (MIF) where the dilution factor was 100 as recommended by the manufacture. A volume of total 50 µl of samples and dilution buffer were added in each plate well and incubated for 1 h and then washed 3 times. Secondary detection antibodies conjugated electrochemiluminescence labels were added and plates were incubated for 1 h. The plates were washed 3 times with washing buffer and then 150 µl MSD GOLD read buffer was added to each well and immediately the plate was inserted to MESO QUICKPLEX SQ 120 instrument for analysis of the 71 proteins. Data were collected and analysed using DISCOVERY WORKBENCH data analysis software. The light intensity of all the different proteins examined was converted into concentrations (pg/ml).

### Statistical analysis

Descriptive data are presented as mean and standard deviation (SD) or median and interquartile range (IQR). Differences in background variables were tested with Mann–Whitney *U* test, repeated measurement analysis of variance (ANOVA) was applied with Bonferroni adjustments for *post hoc* testing. The statistical analyses were performed using Statistica version 13 (StatSoft, Oklahoma, USA).

To investigate the possible differences in the whole protein panels, the multidimensional datapoints were visualized with the help of Uniform Manifold Approximation and Projection (UMAP) and using the two first components from Principal Component Analysis (PCA). The correlations between the different proteins in stimulated and unstimulated saliva samples were estimated and visualized, using an ANCOVA model to take into consideration the intra-person correlation inherent to repeated measures.

For longitudinal expressions analysis, proteins values were standardized and collapsed to its timepoint and sample specific means. This resulted in one datapoint for each protein, saliva collection method, and timepoint. These mean curves were then clustered using k means clustering with three centres, separately for the stimulated and unstimulated samples. The means of the resulting clusters were then plotted longitudinally. The confidence interval bars presented in the graphs are the 95% normal approximation intervals, calculated from the means of the proteins in the cluster at the timepoint in question.

The differences between clusters for each time point were tested with a linear regression model, using the mean protein values and subsetting the data by timepoint. Within cluster differences between timepoints were tested with a linear Generalized Estimation Equations (GEE) model where the cluster was the protein, measured repeatedly over time. Exchangeable correlation structure was used.

The clustering analysis was then repeated including the plasma samples, focusing on the first and last timepoints Due to the small sample size, there is inherently a lot of uncertainty in any estimates. To further investigate the separation between stimulated and unstimulated samples, a Least Absolute Shrinkage and Selection Operator (LASSO) model was used. A logistic regression LASSO was estimated and leave-one-out cross validated to gain understanding of the predictive accuracy, followed by a ROC curve plot.

Analysis was conducted using R, version 4.1.2 (R Core Team (2021), Vienna, Austria. Statistical significance was set at the 95% level.

## Results

### Descriptive data

Descriptive data for all participants in the study are presented in Table 1. Participants included in the study reported no perceived signs of psychological distress and they were all non-smokers.

**Table 1:** Background data of age, anthropometric data, psychological distress, jaw functional limitations, and salivary flow of all healthy participants in the study (*n* = 10) and subdivided by gender.

Variable	All (*n* = 10)	Males (*n* = 5)	Females (*n* = 5)	*P*-value
Age (Years)	26.3 ± 3.1	26.1 ± 3.2	26.4 ± 3.4	1.000
Body Mass Index (kg/m^2^)	21.6 ± 3.0	21.5 ± 2.8	21.6 ± 3.6	1.000
Number of teeth	31 (3)	30 (1)	32 (0)	0.256
PHQ-9 Score	2.5 (4)	0 (5)	2 (2)	0.671
PHQ-15 Score	4 (3)	1 (4)	4 (2)	0.083
GAD-7 Score	1 (4)	0 (2)	1 (3)	0.449
PSS-10 Score	7.5 (6)	8 (3)	7 (6)	0.917
JFLS Score	0 (0)	0 (0)	0 (0)	0.424

Data are presented as mean ± SD or median (IQR).

*n*, number of subjects in each group; PHQ, The Patient Health Questionnaire; GAD, Generalized Anxiety Disorder; PSS, Perceived Stress Scale; JFLS, Jaw Functional Limitation Scale.

The saliva secretion rate between collection points differed significantly for both stimulated (*F* = 6, 25; *P* < 0.001), and unstimulated saliva (*F* = 6.84; *P* < 0.0001) and showed an increasing rate during the day (Table 2). Unstimulated saliva showed higher secretion rate at 07.30 pm compared to the fasting sample at 07.30 am (*P* = 0.0001), while stimulated saliva showed significantly higher flow rate at 4.30 pm compared to 7.30 am (*P* = 0.002) after adjustment for multiple comparison. Salivary flow differed between simulated and unstimulated saliva but was not affected by sex (Mann–Whitney *U* Test; *P* > 0.05).

**Table 2:** Average salivary flow ± SD measured during the collection of unstimulated and stimulated saliva at various time points in a group of 10 healthy individuals.

Collection time	Unstimulated whole saliva ml/min	Stimulated whole saliva ml/min
7:30 am	0.217 ± 0.149	1.810 ± 0.905
10:30 am	0.294 ± 0.179	2.106 ± 0.984
1:30 pm	0.338 ± 0.178	2.283 ± 1.080
4:30 pm	0.357 ± 0.211	2.581 ± 1.449^#^
7:30 pm	0.536 ± 0.321*	2.566 ± 1.450

For both unstimulated and stimulated saliva, the flow rates exhibited significant variations throughout the day (ANOVA, *P* < 0.001).

*Significantly higher compared to at 7.30 am (Bonferroni test, *P* < 0.001). **^#^**Significantly higher than at 7.30 am (Bonferroni test, *P* = 0.002).

### Diurnal variation of mediators

Each mediator in saliva were plotted separately for each participant (*n* = 10) along with the mean value among the cohort by sample type and time point (Supplementary Figure 1). The raw protein data was scaled for comparability and aggregated into sample- and time-specific mean values. These values were then plotted together.

The PCA and UMAP analyses showed that the mediators in plasma and saliva clustered very differently (Figure 1). However, even the two saliva sample types clustered clearly with both the methods. The time of sampling or the gender of the participants did not seem to be as important to the clustering as the sample type (Supplementary Figures 2A,B).

**Figure 2:**
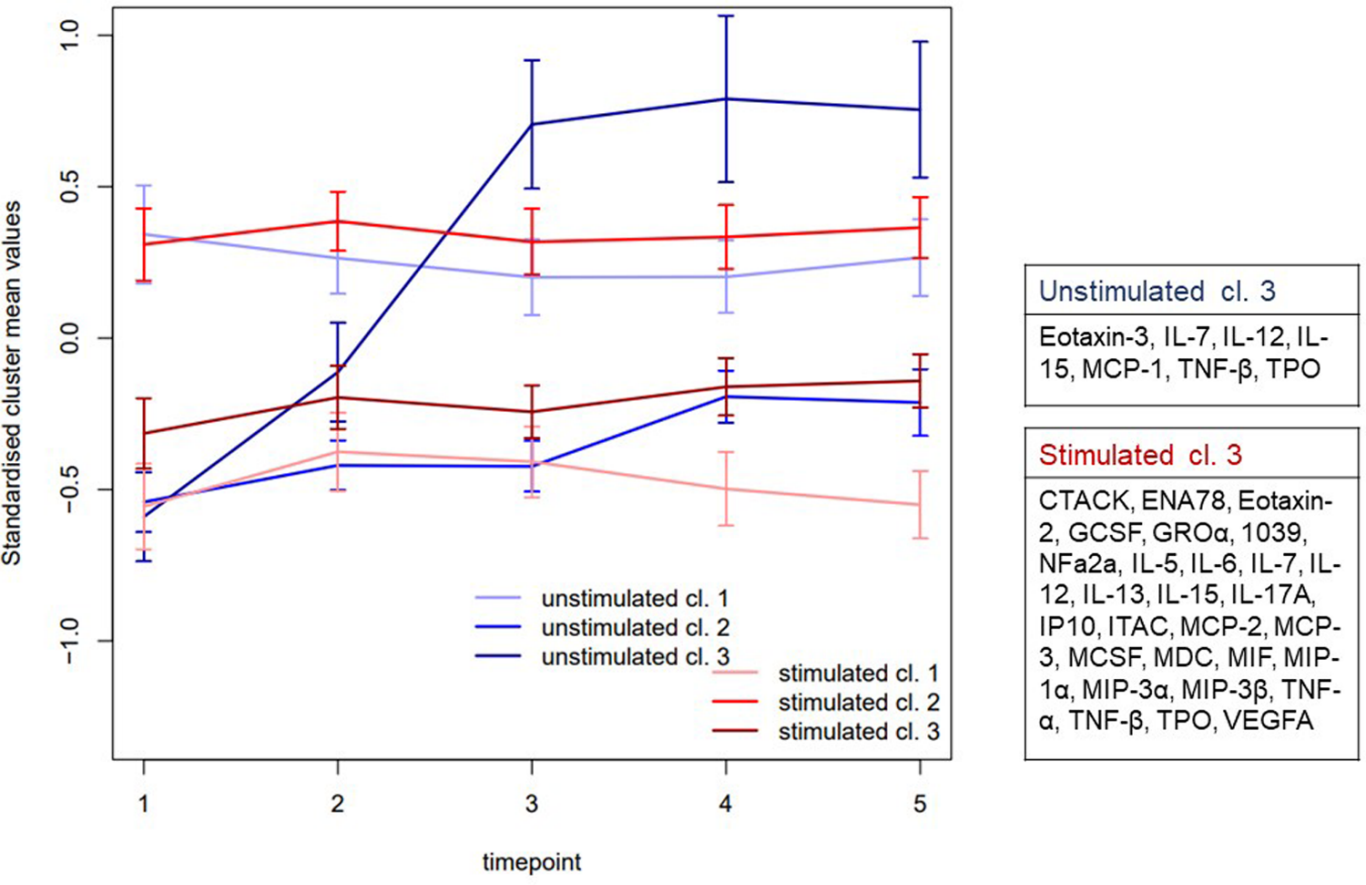
Clustering the mean values. The standardized cluster means and (95% CI) of proteins in unstimulated and stimulated saliva at the different time points (1 = 7.30 am, 2 = 10.30 am, 3 = 1.30 pm, 4 = 4.30 pm, 5 = 7.30 pm). The proteins were reduced to their time and sample specific mean values. This aggregated data was then used to cluster the proteins to three clusters in both samples respectively. Three clusters (cl.) were used as it was considered sufficient to display the different aspects of the two samples (Supplementary Table 1). While there were no differences over time in stimulated whole saliva (red), unstimulated whole saliva (blue) displayed significant fluctuating pattern in two of three cl. In whole unstimulated saliva cl. 3 in showed a significant increase throughput the day (*P* < 0.001), while cl. 2 showed a significant increase between baseline and time point 4 (*P* = 0.001) as well as time point 5 (*P* = 0.000).

Clustering the proteins in each sample type respectively (Supplementary Table 1) identified three clusters (cl.) This was considered sufficient to display the different aspects of the two saliva types (Figure 2). In stimulated saliva the mediators could be categorized in low (cl. 1, mean: −0.556, 95% Cl 0.063, 78.843), middle (cl. 3, mean: −0.293, 95% Cl 0.083, 12.540) and high (cl. 2 mean: 0.307, 95% Cl 0.069, 19.824) concentration group at baseline. These groups showed overall a constant pattern, low longitudinal variability and were essentially stable during the day (*P* > 0.05). However, unstimulated whole saliva showed more fluctuating pattern where two of three clusters displayed significant diurnal changes. Cl. 3 showed a significant increase throughput the day (*P* < 0.001), while cl. 2 showed a significant increase at 4:30 pm (mean: 0.373, 95% Cl: 0.114, 10.593, *P* = 0.001) and 7:30 pm (mean: 0.354, 95% Cl: 0.096, 13.497, *P* = 0.000) compared to morning sample.

The sample specific mean values were also reduced and compared to plasma samples between the first (T1) and last sample (T5) (Supplementary Figure 3).

## Discussion

In recent years, investigating the diurnal variation of inflammatory mediators has gained significant attention due to its potential implications for understanding the circadian rhythm of immune responses (28, 29). The present study capitalizes on the growing recognition of salivás diagnostic potential as a valuable source of molecular information reflecting health status (3, 4).

The study showed clustering patterns in the inflammatory panel between plasma and saliva samples (Figure 1) suggesting that these biofluids provide complementary information. Even within the two saliva sample types, clustering was evident, suggesting differences in mediator expression between unstimulated and stimulated saliva. Notably, while the sampling time and participant sex (Supplementary Figure 1) did not strongly influence clustering, sample type emerged as a key factor (Figure 1). These results align with other studies showing that the sample type have stronger impact on the protein expression than sex and underscore the need for standardized saliva collection methods in future studies to minimize variability and ensure comparability of results (18, 19, 30).

There are different methods described to collect saliva. Most studies focus on unstimulated whole saliva via passive drooling, considered the “gold standard” of saliva collection. This method avoids oral stimulation, which can alter mediator compositions and concentrations (16, 21). Other studies uses stimulated whole saliva (31, 32) or “semi-stimulated saliva” (15). Currently, only a handful studies have compared proteins and inflammatory substances between different salivary collection methods (19–21, 33, 34). In previous studies, we demonstrated significant variation between different saliva collection methods regarding protein expressed at high abundant (ng/µg level) and allogenic peptides (19, 20). These variations were notable between the different collection methods, with stimulated whole saliva emerging as the most reliable method due to its low variability and higher protein expression (3), in line with current findings.

Foratori-Junior et al., (33) compared the proteomic profile of unstimulated and stimulated saliva in healthy pregnant women and found that stimulation decreased proteins involved in immune response and inflammation (33). A similar trend could also be observed for certain substances in this study (Supplementary Figure 3). For example, cutaneous T cell-attracting chemokine (CTACK); Epithelial-Derived Neutrophil-Activating peptide 78 (ENA78); Eotaxin; Erythropoietin (EPO); IL-7; IL-13; IL-17A; Monocyte Chemoattractant Protein (MCP); Macrophage Colony-Stimulating Factor (MCSF), and Thymus Activation-Regulated Chemokine (TARC) decreased with saliva stimulation, while others like e.g., IL-17B; IL-17C; IL-17D; IL-17E; L-17F; IL-1α; IL-1β; IL-2; IL-3; IL-21; IL-22; IL-23 increased. Several markers such as Granulocyte Colony-Stimulating Factor (GCSF); IL-6; MIF; TNF-α were unaffected by stimulation. The differing stability patterns between unstimulated and stimulated saliva suggest that certain mediators may be more reliably detected in one type of saliva over the other. However, the sample size in this pilot study is too small to draw significant conclusions regarding individual markers. It should also be noted that previous studies have primarily focused on proteins that occur in high concentration in saliva (19, 20, 33), rather than on those present at picomolar levels, such as cytokines, chemokines and growth factors.

When comparing the concentration of mediators in saliva to that in plasma, disparities became evident, raising questions about their ability to adequately reflect systemic biological events. While, for example, salivary cortisol levels may effectively mirror systemic levels (35), salivary levels of some inflammatory meditators, such as C-reactive protein (CRP) and IL-6 lack significant correlation with plasma levels (15, 21, 36). This discrepancy can be attributed to the distinct environmental niche of the oral cavity, where local immune processes play a substantial role. Cytokine levels in saliva associated with gingival inflammation and periodontitis (37, 38) have been shown to primarily reflect localized rather than systemic immune responses. Consequently, the use of salivary cytokine levels as general surrogate markers for assessing systemic immune responses should be approached cautiously (15).

We aggregated protein data to their respective mean values based on time and sample type, then used this dataset to cluster each sample type separately. Our analysis revealed three distinct clusters that effectively captured the diverse aspects of both stimulated and unstimulated saliva (Figure 2). The patterns observed in the samples collected in unstimulated saliva displayed greater variability compared to the stimulated samples. In stimulated saliva, proteins were clustered into low, middle, and high concentration groups between time-point, showing consistent patterns with low variability, indicating stability throughout the day. In contrast, the unstimulated whole saliva displayed more fluctuating patterns, with one specific cluster showing a significant increase in concentration throughout the day (cl. 3) and another cluster (cl. 2) showing a significant increase in the afternoon. These diurnal patterns suggest that salivary collection methods and time of the day could influence the detection and interpretation of some mediators. The mediators showing increased expression from morning to evening included cytokines IL-7, IL-12, and IL-15, TNF-β, Thrombopoietin (TPO) and chemokines Eotaxin-3 and MCP-1. Despite their role in immune repose and regulation (39), there are no previous studies regarding the salivary circadian rhythm in healthy individuals of these substances. Dalgar and co-authors studied the diurnal variation of MCP-1 in plasma in healthy individuals compared to patients with post-traumatic stress syndrome and found that MCP-1 exhibited a peak upon awaking, followed by a decrease during the day (40). Studies have also investigated the diurnal variations in saliva for other cytokines, such as IL-1β (41, 42), IL-6 (24, 41, 43) and TNF (42). Similar trends have also been observed in these studies, with elevated levels typically occurring upon awakening and during the daylight hours, followed by a decline throughout the day. These results are in contrast with the trend observed in cluster 3. It should be noted, however, that all these previous studies have employed different collection methods, different time-points with a varying number of samples taken, and differing methodologies. This makes direct comparisons with our study challenging. In our study, the first sample was collected at 7.30 am, i.e., between 0.5 and 3 h after awaking, and the last one was collected 7.30 pm, which means that we could not observe the diurnal variation upon awakening, before bedtime, or during the night.

A strength in our study design was that stimulated and unstimulated whole saliva was consistently collected each third hour under exactly the same clinical condition. Anamnesis and careful oral and dental examinations were conducted to ensure that participants were healthy and without any signs of local inflammation. Strict inclusion criteria were implemented to reduce the influence of external and internal factors on salivary flow, secretion, and content. Furthermore, the participants were closely matched in age to minimize the impact of age-related factors on flow rate and mediator expression (44). Nonetheless, our findings should be interpreted within the context of certain limitations. The study was conducted in healthy young adults and included a small number of participants. Different age groups were not taken into considered due to the potential for age-related variability (45). The absence of samples during the night, immediately before sleep, or upon awakening may have limited our ability to observe certain important fluctuations influenced by the sleep-wake phase.

Despite the limitations, this study offers valuable insights into the diurnal variation of salivary cytokines, chemokines and growth factors, highlighting the importance of collection method in biomarker analysis. The distinct clustering patterns observed between unstimulated and stimulated saliva emphasize the need to consider the type of collection method when interpreting biomarker data. Our findings reveled that salivary cytokines, chemokines, and growth factors follow diurnal pattern, with stimulated saliva showing more stable and consistent profiles compared to unstimulated saliva that exhibited a greater variability for certain mediators. These results suggest that time of the day could influence the detection and interpretation of inflammatory markers and collecting saliva samples at consistent times across participants will help control for the natural fluctuations in salivary composition. The results encourage further exploration of salivary diagnostics, particularly in understanding circadian rhythms and localized immune responses.

## Data Availability

The datasets presented in this article are not readily available because the data contains information that could compromise the privacy of research participants. Requests to access the datasets should be directed to the corresponding author.

## Supplementary material

Supplementary Table 1 The following analytes were measured by a panel of 71 pro- and anti-inflammatory proteins from U-PLEX, Meso Scale Discovery, Maryland, USA. The proteins were condensed to their mean values specific to both time points and samples. Subsequently, this consolidated dataset was employed to categorize the proteins into three clusters for each sample type (cl 1–3). The figure illustrates the group belonging for the cytokines and chemokines in the analysis.

Supplementary Figure 1 Uniform Manifold Approximation and Projection (UMAP) is used to graphically cluster all samples based on biological fluid (A) and sex (B). The figure is indicative that plasma and saliva clusters different (A). The sex of the participants does not seem to affect the clustering (B).

Supplementary Figure 2 Figure illustrate the mean differences between the three clusters in plasma, unstimulated and stimulated whole saliva between morning (T1) and evening (T5) samples.

Supplementary Figure 3 Diurnal variation of cytokines, chemokines and growth factors graphed individually for each participant (*n* = 10) in unstimulated (blue) and stimulated (red) whole saliva. The means of each protein by sample type and time are visualised as a bold line.

## References

[1] Drobitch RK Svensson CK. Therapeutic drug monitoring in saliva. An update. Clin Pharmacokinet. (1992) 23(5):365–79. 10.2165/00003088-199223050-00003 1478004

[2] Yoshizawa JM Schafer CA Schafer JJ Farrell JJ Paster BJ Wong DT. Salivary biomarkers: toward future clinical and diagnostic utilities. Clin Microbiol Rev. (2013) 26(4):781–91. 10.1128/CMR.00021-13 24092855 PMC3811231

[3] Jasim H. Topical review—salivary biomarkers in chronic muscle pain. Scand J Pain. (2023) 23(1):3–13. 10.1515/sjpain-2022-0112 36228098

[4] Liu J Duan Y. Saliva: a potential media for disease diagnostics and monitoring. Oral Oncol. (2012) 48(7):569–77. 10.1016/j.oraloncology.2012.01.021 22349278

[5] Hu S Arellano M Boontheung P Wang J Zhou H Jiang J Salivary proteomics for oral cancer biomarker discovery. Clin Cancer Res. (2008) 14(19):6246–52. 10.1158/1078-0432.CCR-07-5037 18829504 PMC2877125

[6] Hu S Wang J Meijer J Ieong S Xie Y Yu T Salivary proteomic and genomic biomarkers for primary sjogren’s syndrome. Arthritis Rheum. (2007) 56(11):3588–600. 10.1002/art.22954 17968930 PMC2856841

[7] Dakovic D Colic M Cakic S Mileusnic I Hajdukovic Z Stamatovic N. Salivary interleukin-8 levels in children suffering from type 1 diabetes mellitus. J Clin Pediatr Dent. (2013) 37(4):377–80. 10.17796/jcpd.37.4.l135531h4542gj66 24046985

[8] Malamud D. Oral diagnostic testing for detecting human immunodeficiency virus-1 antibodies: a technology whose time has come. Am J Med. (1997) 102(4a):9–14. 10.1016/S0002-9343(97)00032-6 9217633

[9] Ochnio JJ Scheifele DW Ho M Mitchell LA. New, ultrasensitive enzyme immunoassay for detecting vaccine- and disease-induced hepatitis A virus-specific immunoglobulin G in saliva. J Clin Microbiol. (1997) 35(1):98–101. 10.1128/jcm.35.1.98-101.1997 8968887 PMC229518

[10] Emmons W. Accuracy of oral specimen testing for human immunodeficiency virus. Am J Med. (1997) 102(4a):15–20. 10.1016/S0002-9343(97)00033-8 9217634

[11] Adam DJ Milne AA Evans SM Roulston JE Lee AJ Ruckley CV Serum amylase isoenzymes in patients undergoing operation for ruptured and non-ruptured abdominal aortic aneurysm. J Vasc Surg. (1999) 30(2):229–35. 10.1016/S0741-5214(99)70132-1 10436442

[12] Lee YH Wong DT. Saliva: an emerging biofluid for early detection of diseases. Am J Dent. (2009) 22(4):241–8. 19824562 PMC2860957

[13] Slavish DC Graham-Engeland JE Smyth JM Engeland CG. Salivary markers of inflammation in response to acute stress. Brain Behav Immun. (2015) 44:253–69. 10.1016/j.bbi.2014.08.008 25205395 PMC4275319

[14] Herder C de Las Heras Gala T Carstensen-Kirberg M Huth C Zierer A Wahl S Circulating levels of interleukin 1-receptor antagonist and risk of cardiovascular disease: meta-analysis of six population-based cohorts. Arterioscler, Thromb, Vasc Biol. (2017) 37(6):1222–7. 10.1161/ATVBAHA.117.309307 28428221

[15] Szabo YZ Slavish DC. Measuring salivary markers of inflammation in health research: a review of methodological considerations and best practices. Psychoneuroendocrinology. (2021) 124:105069. 10.1016/j.psyneuen.2020.105069 33316694 PMC8412951

[16] Diesch T Filippi C Fritschi N Filippi A Ritz N. Cytokines in saliva as biomarkers of oral and systemic oncological or infectious diseases: a systematic review. Cytokine. (2021) 143:155506. 10.1016/j.cyto.2021.155506 33846070

[17] Al Kawas S Rahim ZH Ferguson DB. Potential uses of human salivary protein and peptide analysis in the diagnosis of disease. Arch Oral Biol. (2012) 57(1):1–9. 10.1016/j.archoralbio.2011.06.013 21774913

[18] Jasim H Carlsson A Gerdle B Ernberg M Ghafouri B. Diurnal variation of inflammatory plasma proteins involved in pain. Pain Rep. (2019) 4(5):e776. 10.1097/PR9.0000000000000776 31875183 PMC6882578

[19] Jasim H Olausson P Hedenberg-Magnusson B Ernberg M Ghafouri B. The proteomic profile of whole and glandular saliva in healthy pain-free subjects. Sci Rep. (2016) 6:39073. 10.1038/srep39073 27976689 PMC5157045

[20] Jasim H Carlsson A Hedenberg-Magnusson B Ghafouri B Ernberg M. Saliva as a medium to detect and measure biomarkers related to pain. Sci Rep. (2018) 8(1):3220. 10.1038/s41598-018-21131-4 29459715 PMC5818517

[21] Williamson S Munro C Pickler R Grap MJ Elswick RK Jr. Comparison of biomarkers in blood and saliva in healthy adults. Nurs Res Pract. (2012) 2012:246178. 10.1155/2012/246178 22619709 PMC3350846

[22] Baxter M Ray DW. Circadian rhythms in innate immunity and stress responses. Immunology. (2020) 161(4):261–7. 10.1111/imm.13166 31820826 PMC7692257

[23] Jasim H Louca S Christidis N Ernberg M. Salivary cortisol and psychological factors in women with chronic and acute oro-facial pain. J Oral Rehabil. (2014) 41(2):122–32. 10.1111/joor.12118 24313837

[24] Izawa S Miki K Liu X Ogawa N. The diurnal patterns of salivary interleukin-6 and C-reactive protein in healthy young adults. Brain Behav Immun. (2013) 27(1):38–41. 10.1016/j.bbi.2012.07.001 22796263

[25] Tonetti MS Greenwell H Kornman KS. Staging and grading of periodontitis: framework and proposal of a new classification and case definition. J Periodontol. (2018) 89(Suppl 1):S159–s72. 10.1002/JPER.18-0006 29926952

[26] Nederfors T. Xerostomia and hyposalivation. Adv Dent Res. (2000) 14:48–56. 10.1177/08959374000140010701 11842923

[27] Schiffman E Ohrbach R Truelove E Look J Anderson G Goulet JP Diagnostic criteria for temporomandibular disorders (DC/TMD) for clinical and research applications: recommendations of the international RDC/TMD consortium network* and orofacial pain special interest groupdagger. J Oral Facial Pain Headache. (2014) 28(1):6–27. 10.11607/jop.1151 24482784 PMC4478082

[28] Curtis AM Bellet MM Sassone-Corsi P O'Neill LA. Circadian clock proteins and immunity. Immunity. (2014) 40(2):178–86. 10.1016/j.immuni.2014.02.002 24560196

[29] Orozco-Solis R Aguilar-Arnal L. Circadian regulation of immunity through epigenetic mechanisms. Front Cell Infect Microbiol. (2020) 10:96. 10.3389/fcimb.2020.00096 32232012 PMC7082642

[30] Xiao X Liu Y Guo Z Liu X Sun H Li Q Comparative proteomic analysis of the influence of gender and acid stimulation on normal human saliva using LC/MS/MS. Proteom Clin Appl. (2017) 11(7–8). 10.1002/prca.201600142 28198151

[31] Jasim H Ernberg M Carlsson A Gerdle B Ghafouri B. Protein signature in Saliva of temporomandibular disorders myalgia. Int J Mol Sci. (2020) 21(7):2569. 10.3390/ijms21072569 32272779 PMC7177369

[32] Jasim H Ghafouri B Gerdle B Hedenberg-Magnusson B Ernberg M. Altered levels of salivary and plasma pain related markers in temporomandibular disorders. J Headache Pain. (2020) 21(1):105. 10.1186/s10194-020-01160-z 32842964 PMC7449051

[33] Foratori-Junior GA Ventura TMO Grizzo LT Jesuino BG Castilho A Buzalaf MAR Is there a difference in the proteomic profile of stimulated and unstimulated Saliva samples from pregnant women with/without obesity and periodontitis? Cells. (2023) 12(10). 10.3390/cells12101389 37408223 PMC10216649

[34] Jasim H Ghafouri B Carlsson A Hedenberg-Magnusson B Ernberg M. Daytime changes of salivary biomarkers involved in pain. J Oral Rehabil. (2020) 47(7):843–50. 10.1111/joor.12977 32277715

[35] Poll EM Kreitschmann-Andermahr I Langejuergen Y Stanzel S Gilsbach JM Gressner A Saliva collection method affects predictability of serum cortisol. Clin Chim Acta. (2007) 382(1-2):15–9. 10.1016/j.cca.2007.03.009 17449021

[36] Sjögren E Leanderson P Kristenson M Ernerudh J. Interleukin-6 levels in relation to psychosocial factors: studies on serum, saliva, and *in vitro* production by blood mononuclear cells. Brain Behav Immun. (2006) 20(3):270–8. 10.1016/j.bbi.2005.08.001 16183246

[37] Kc S Wang XZ Gallagher JE. Diagnostic sensitivity and specificity of host-derived salivary biomarkers in periodontal disease amongst adults: systematic review. J Clin Periodontol. (2020) 47(3):289–308. 10.1111/jcpe.13218 31701554

[38] Kawamoto D Amado PPL Albuquerque-Souza E Bueno MR Vale GC Saraiva L Chemokines and cytokines profile in whole saliva of patients with periodontitis. Cytokine. (2020) 135:155197. 10.1016/j.cyto.2020.155197 32707521

[39] Briukhovetska D Dörr J Endres S Libby P Dinarello CA Kobold S. Interleukins in cancer: from biology to therapy. Nat Rev Cancer. (2021) 21(8):481–99. 10.1038/s41568-021-00363-z 34083781 PMC8173513

[40] Dalgard C Eidelman O Jozwik C Olsen CH Srivastava M Biswas R The MCP-4/MCP-1 ratio in plasma is a candidate circadian biomarker for chronic post-traumatic stress disorder. Transl Psychiatry. (2017) 7(2):e1025. 10.1038/tp.2016.285 28170001 PMC5438024

[41] Ghazali NB Steele M Koh D Idris A. The diurnal pattern of salivary IL-1β in healthy young adults. Int J Adolesc Med Health. (2017) 31(5). 10.1515/ijamh-2017-0058 28782347

[42] Reinhardt ÉL Fernandes P Markus RP Fischer FM. Night work effects on salivary cytokines TNF, IL-1β and IL-6. Chronobiol Int. (2019) 36(1):11–26. 10.1080/07420528.2018.1515771 30230913

[43] Hori H Izawa S Yoshida F Kunugi H Kim Y Mizukami S Association of childhood maltreatment history with salivary interleukin-6 diurnal patterns and C-reactive protein in healthy adults. Brain Behav Immun. (2022) 101:377–82. 10.1016/j.bbi.2022.01.020 35093493

[44] Fleissig Y Reichenberg E Redlich M Zaks B Deutsch O Aframian DJ Comparative proteomic analysis of human oral fluids according to gender and age. Oral Dis. (2010) 16(8):831–8. 10.1111/j.1601-0825.2010.01696.x 20561216

[45] Prodan A Brand HS Ligtenberg AJ Imangaliyev S Tsivtsivadze E van der Weijden F Interindividual variation, correlations, and sex-related differences in the salivary biochemistry of young healthy adults. Eur J Oral Sci. (2015) 123(3):149–57. 10.1111/eos.12182 25809904

