# Supplementary figures and images for: The diurnal pattern of cytokines, chemokines and growth factors in human saliva—a pilot study
Source: Front Dent Med. 2024 Nov 20;5:1420081. doi: 10.3389/fdmed.2024.1420081 (PMC11797753; doi:10.3389/fdmed.2024.1420081)

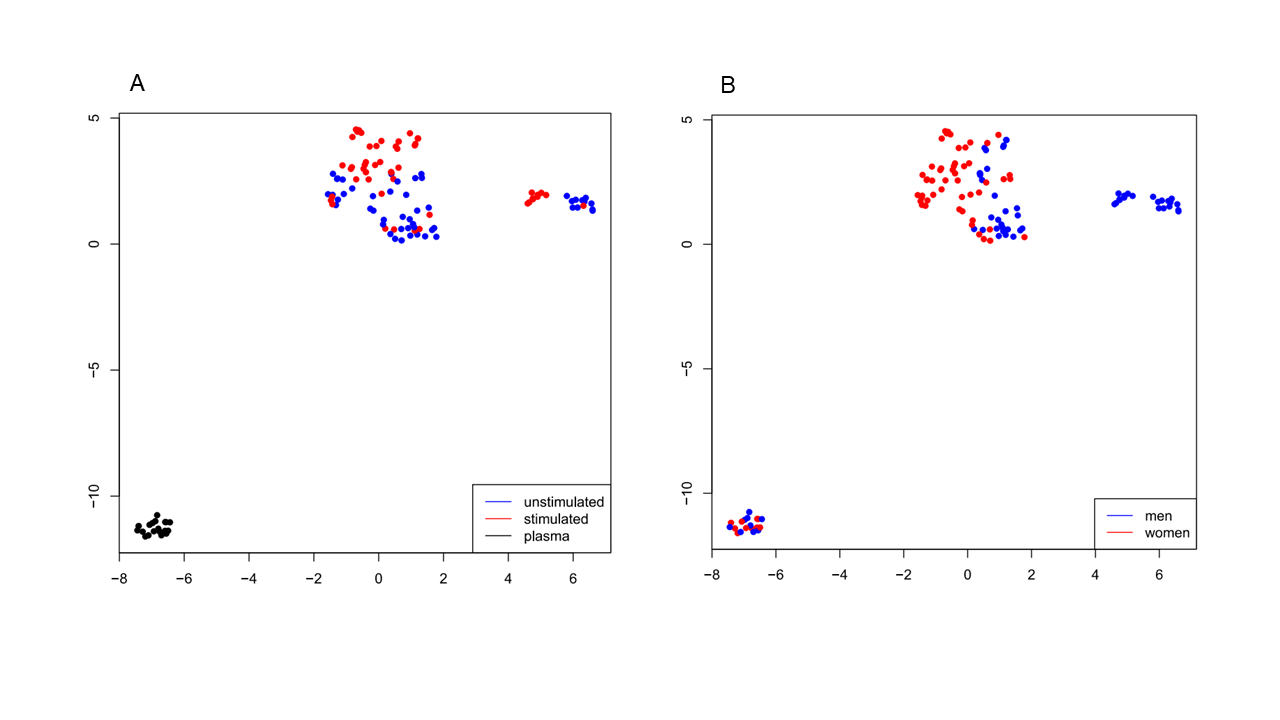

Supplement: Supplementary Figure 1 — Uniform Manifold Approximation and Projection (UMAP) is used to graphically cluster all samples based on biological fluid (A) and sex (B). The figure is indicative that plasma and saliva clusters different (A). The sex of the participants does not seem to affect the clustering (B). [file Image1.tif]

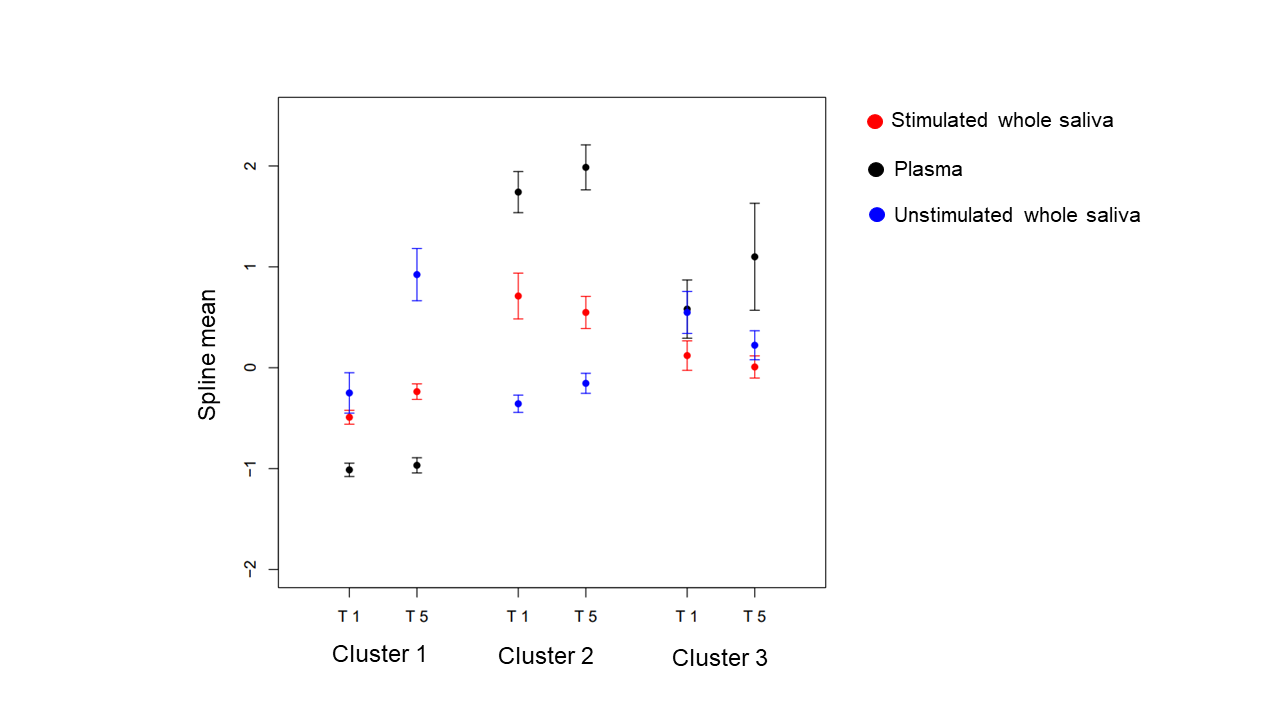

Supplement: Supplementary Figure 2 [file Image2.tif]
