# Supplementary material for: The diurnal pattern of cytokines, chemokines and growth factors in human saliva—a pilot study
Source: Front Dent Med. 2024 Nov 20;5:1420081. doi: 10.3389/fdmed.2024.1420081 (PMC11797753; doi:10.3389/fdmed.2024.1420081)
Supplement: Supplementary Figure 3 — Diurnal variation of cytokines, chemokines and growth factors graphed individually for each participant (n = 10) in unstimulated (blue) and stimulated (red) whole saliva. The means of each protein by sample type and time are visualised as a bold line. [file Datasheet1.pdf]

Supplementary Table 1

Protein	Unstimulated saliva	Stimulated saliva
CTACK	cl. 1	cl. 3
ENA78	cl. 1	cl. 3
Eotaxin	cl. 1	cl. 1
Eotaxin-2	cl. 1	cl. 3
Eotaxin-3	cl. 3	cl. 1
EPO	cl. 1	cl. 1
FLT3L	cl. 2	cl. 2
Fractalkine	cl. 1	cl. 1
GCSF	cl. 1	cl. 3
GMCSF	cl. 2	cl. 2
GROα	cl. 1	cl. 3
1039	cl. 1	cl. 3
INFa2a	cl. 1	cl. 3
IFNβ	cl. 2	cl. 2
IFNg	cl. 2	cl. 2
IL-10	cl. 2	cl. 2
IL-12	cl. 3	cl. 3
IL-12p70	cl. 2	cl. 2
IL-13	cl. 1	cl. 3
IL-15	cl. 3	cl. 3
IL-16	cl. 2	cl. 2
IL-17A	cl. 1	cl. 3
IL-17AF	cl. 1	cl. 1
IL-17B	cl. 2	cl. 2
IL-17C	cl. 2	cl. 2
IL-17D	cl. 2	cl. 2
IL-7E/IL-25	cl. 2	cl. 2
IL-17F	cl. 2	cl. 2
IL-18	cl. 2	cl. 2
IL-1RA	cl. 2	cl. 2
IL-1α	cl. 1	cl. 2
IL-1β	cl. 2	cl. 2
IL-2	cl. 2	cl. 2
IL-21	cl. 2	cl. 2
IL-22	cl. 2	cl. 2
IL-23	cl. 2	cl. 2
IL-27	cl. 2	cl. 2
IL-29/IFNL1	cl. 2	cl. 2
IL-2RA	cl. 2	cl. 2
IL-3	cl. 2	cl. 2
IL-31	cl. 2	cl. 2
IL-33	cl. 2	cl. 2
IL-4	cl. 2	cl. 2
IL-5	cl. 1	cl. 3
IL-6	cl. 1	cl. 3
IL-7	cl. 3	cl. 3
IL-8	cl. 2	cl. 2
IL-9	cl. 2	cl. 2
IP10	cl. 1	cl. 3
ITAC	cl. 1	cl. 3

MCP-1	cl. 3	cl. 1
MCP-2	cl. 1	cl. 3
MCP-3	cl. 1	cl. 3
MCP-4	cl. 1	cl. 1
MCSF	cl. 1	cl. 3
MDC	cl. 1	cl. 3
MIF	cl. 1	cl. 3
MIP-1α	cl. 1	cl. 3
MIP-1β	cl. 2	cl. 2
MIP-3α	cl. 1	cl. 3
MIP-3β	cl. 1	cl. 3
MIP-5	cl. 1	cl. 1
SDF-1α	cl. 2	cl. 2
TARC	cl. 1	cl. 1
TNF-α	cl. 1	cl. 3
TNF-β	cl. 3	cl. 3
TPO	cl. 3	cl. 3
TRAIL	cl. 1	cl. 1
TSLP	cl. 2	cl. 2
VEGFA	cl. 1	cl. 3
YKL40	cl. 2	cl. 2
